# Red emission fluorescent probes for visualization of monoamine oxidase in living cells

**DOI:** 10.1038/srep31217

**Published:** 2016-08-08

**Authors:** Ling-Ling Li, Kun Li, Yan-Hong Liu, Hao-Ran Xu, Xiao-Qi Yu

**Affiliations:** 1 Key Laboratory of Green Chemistry and Technology, Ministry of Education,College of Chemistry, Sichuan University, No. 29, Wangjiang Road, Chengdu, 610064, P. R. China

## Abstract

Here we report two novel red emission fluorescent probes for the highly sensitive and selective detection of monoamine oxidase (MAO) with large Stokes shift (227 nm). Both of the probes possess solid state fluorescence and can accomplish the identification of MAO on test papers. The probe **MAO-Red-1** exhibited a detection limit down to 1.2 μg mL^−1^ towards MAO-B. Moreover, the cleavage product was unequivocally conformedby HPLC and LCMS and the result was in accordance with the proposed oxidative deamination mechanism. The excellent photostability of **MAO-Red-1** was proved both *in vitro* and *in vivo* through fluorescent kinetic experiment and laser exposure experiment of confocal microscopy, respectively. Intracellular experiments also confirmed the low cytotoxity and exceptional cell imaging abilities of **MAO-Red-1**. It was validated both in HeLa and HepG2 cells that **MAO-Red-1** was capable of reporting MAO activity through the variation of fluorescence intensity.

Monoamine oxidase (MAO) is a flavin-dependent mitochondrial enzyme that catalyzes the oxidation of neuroactive and vasoactive amines such as dopamine, norepinephrine, adrenalin, and serotonin^1^,^2^. MAO-A and MAO-B are two isoforms of MAO, with 70% sequence identity and identical FAD cofactors^3^. Despite their resemblances, they also differ in a great many ways, including substrate and inhibitor specificities, and physiological functions^4^. MAO-A mainly metabolizes serotonin and epinephrine, the association between MAO-A and psychiatric disorders, such as social anxiety and depression, is well-documented. As a matter of fact, MAO-A selective inhibitors, take clorgyline and moclobemide for instance, are prescribed as clinical antidepressant agents^5^,^6^,^7^,^8^. MAO-B predominantly catalyzes the oxidative deamination of benzylamine and phenylethylamine and is selectively inhibited by pargyline and selegiline. MAO-B level is found to be associated with neurodegenerative disorders including Alzheimer’s disease and Parkinson’s disease and the selective inhibition of MAO-B is applied to the treatment of those two diseases^9^,^10^,^11^,^12^,^13^,^14^. Together, MAO-A and MAO-B play pivotal roles in maintaining the proper balance of neurotransmitter and other biogenic amines, which are essential for the healthy function of living organisms.

Due to their fundamental roles in sustaining the homeostasis of biogenic amines, MAO-A and MAO-B have drawn many attentions of researchers. Various technologies have been developed for monitoring real-time MAO levels, in an attempt to reveal MAO’s catalytic mechanism, better understand its roles in physiological processes, diagnose irregular MAO level caused diseases, and exploit MAO related drugs^15^,^16^,^17^,^18^,^19^,^20^,^21^. Among these technologies, fluorescence imaging^22^,^23^,^24^ as a powerful non-invasive method with high sensitivity and simplicity has attracted many attentions^7^,^25^,^26^,^27^,^28^. For example, Ahn *et al.* reported two coumarin based fluorophores that emits at 585 nm and achieved two-photon imaging of MAO^29^. Zhu and his colleagues designed a series of fluorescein probes, the maximum emission wavelength of which varies from 530 nm to 570 nm, and were successfully applied to live-cell imaging of MAO activites^30^,^31^. Last year, a group of activity-based MAO-B specific fluorescent probes with emission wavelength up to 530 nm were reported by Yao group^32^. Most of the previously developed MAO probes revealed good fluorescence imaging abilities and sensitivity towards MAO-B, whereas none of them had emission wavelength above 650 nm (Red emission). Fluorescent probes with emission wavelength above 650 nm have a great advantage in avoiding interference from biomolecular autofluorescence, minimizing photo-damage to living cells, and penetrating relatively deeper tissues^33^,^34^,^35^,^36^,^37^,^38^,^39^,^40^. Inspired by these merits, we designed the first red emission fluorescent turn-on probes for the detection of MAO with low detection limit, large Stokes shift, solid-state fluorescence and excellent imaging ability in monitoring MAO activities in living cells. The comprehensive improvement in the fluorescent properties will allow the probes to be widely adopted in biological applications.

Our design strategy for the detection of MAO activities both *in vitro* and *in vivo* is based on the consecutive amine oxidation/β-elimination mechanism as shown in Fig. 1a. We chose (*E*)-2-(2-(4-hydroxystyryl)-4H-chromen-4-ylidene) malononitrile (DCPO) as the fluorescent reporter due to its long emission wavelength and biological compatibility^33^,^40^. Then, we installed two well-known MAO substrates, 3-amino-propoxy group and *N, N*-dimethylpropan-3-amino- propoxy group on the phenolic OH of DCPO to build **MAO-Red-1** and **MAO-Red-2** (Fig. 1b), respectively, which are not fluorescent at longer wavelength in the format of a phenolic ether (OFF state). We envisioned that with the oxidative deamination of MAO, the reactive site will be eliminated, leading to the ether bond cleavage and the release of DCPO. Subsequently, the fluorescence emission of DCPO at about 664 nm will be detected, and the OFF-ON fluorescence change will be observed.

## Results

### Spectra properties of MAO-Red-1 and MAO-Red-2

To explore the sensitivity and selectivity of **MAO-Red-1** and **MAO-Red-2**, we first investigated their fluorescence properties in enzyme assay buffer (100 mM HEPES, pH 7.4 with 5% glycerol and 1% DMSO) at 37 °C. Prior to the treatment of MAO, the solution of **MAO-Red-1** (10 μM) exhibited a fluorescence emission at around 550 nm. However, upon the addition of MAO (10 μg ml^−1^), the fluorescence intensity at 550 nm gradually decreased. Morever, a ~7.2-fold fluorescence increase at 664 nm were observed with MAO-B and ~3.8-fold augment with MAO-A. Additionally, since the maximum absorption wavelength was at 437 nm (Fig. S2), the Stokes shift was therefore calculated to be as large as 227 nm (Fig. 2a), which was highly conducive to minimizing background fluorescence. In addition to that, **MAO-Red-1** displayed a detection limit of 1.2 μg mL^−1^ for MAO-B (Fig. S1), which was relatively low compared with previously reported MAO probes. Moreover, the results demonstrated that both of the probes were capable of detecting MAO activities, **MAO-Red-1** exhibited better reactivity and was therefore further explored in all the subsequent studies. Fluorescence kinetic assays were later carried out between MAO and probes (Fig. 2a, inserted plot), it took less than 20 minutes for **MAO-Red-1** to give a stable readout after the addition of MAO (K_m_ value obtained for **MAO-Red-1** with MAO-B was 270 μM), and about 40 minutes for **MAO-Red-2** with MAO (Fig. S3). **MAO-Red-1** exhibited the greatest signal-to-background ratio towards MAO-B (Fig. 2b), proving its capacity in selective detection of MAO and differentiate MAO-B from MAO-A with a selectivity ratio of about 2.2:1. Unfortunately, the excitation wavelength of the probes was in the UV-vis region, which will lead to the disadvantage in penetrating deep tissues compared with relatively longer excitation wavelength.

To further validate the sensitivity of these probes towards MAO, **MAO-Red-1** and **MAO-Red-2** were respectively incubated with a group of different proteins, including subtilisin, bovine serum albumin (BSA), and pargyline inhibited MAO. No significant enhancement was detected upon addition of these proteins to our probes. We also noticed that **MAO-Red-1**, **MAO-Red-2** and the theoretically product DCPO were all luminescent in the solid state. The maximum fluorescence emission of **MAO-Red-1** and **MAO-Red-2** were measured to be 592 nm and 599 nm, and the quantum yields were 10% and 5%, respectively. While DCPO exhibited a strong emission at about 633 nm (Fig. 2c), suggesting a red shift after the probes were incubated with MAO, which correlated well with the previous fluorescent results conducted in the aqueous solution. On this basis, we dipped filter papers into the methanol solution of **MAO-Red-1**, **MAO-Red-2**, and DCPO (1 mg mL^−1^), respectively. After drying up, DCPO showed obviously different colour from **MAO-Red-1** and **MAO-Red-2** (Fig. 2d) under 365 nm UV light. Given the advantages (i.e. high molar extinction coefficient, larger Stokes shifts, highly modifiable etc.) attributed to solid state fluorescent materials^34^ these novel probes might have the potential to be further employed in the application of MAO test papers.

### Determination of the cleavage product through HPLC and LCMS tests

**Figure 1:**
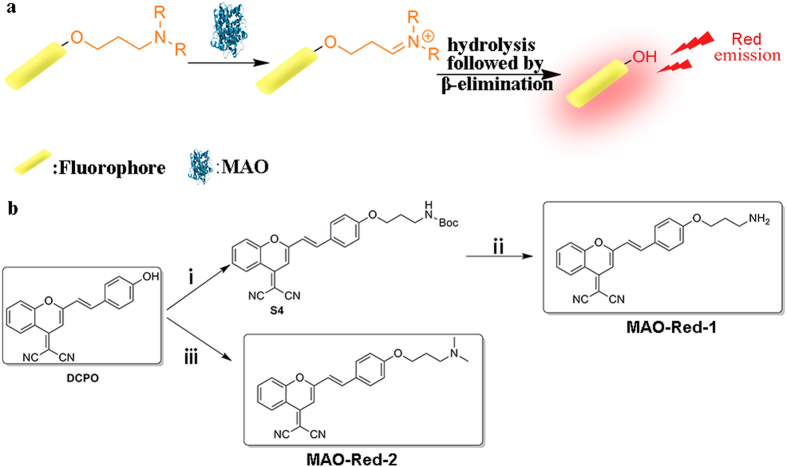
(**a**) The oxidative deamination mechanism of MAO. (**b**) Synthetic route to MAO reporters: **MAO-Red-1** and **MAO-Red-2**. Reagents and conditions: (i) K_2_CO_3_/DMF, r.t, 83%; (ii) HCl/MeOH, r.t, 98%; (iii) K_2_CO_3_/CH_3_COCH_3_, reflux, 61%.

**Figure 2:**
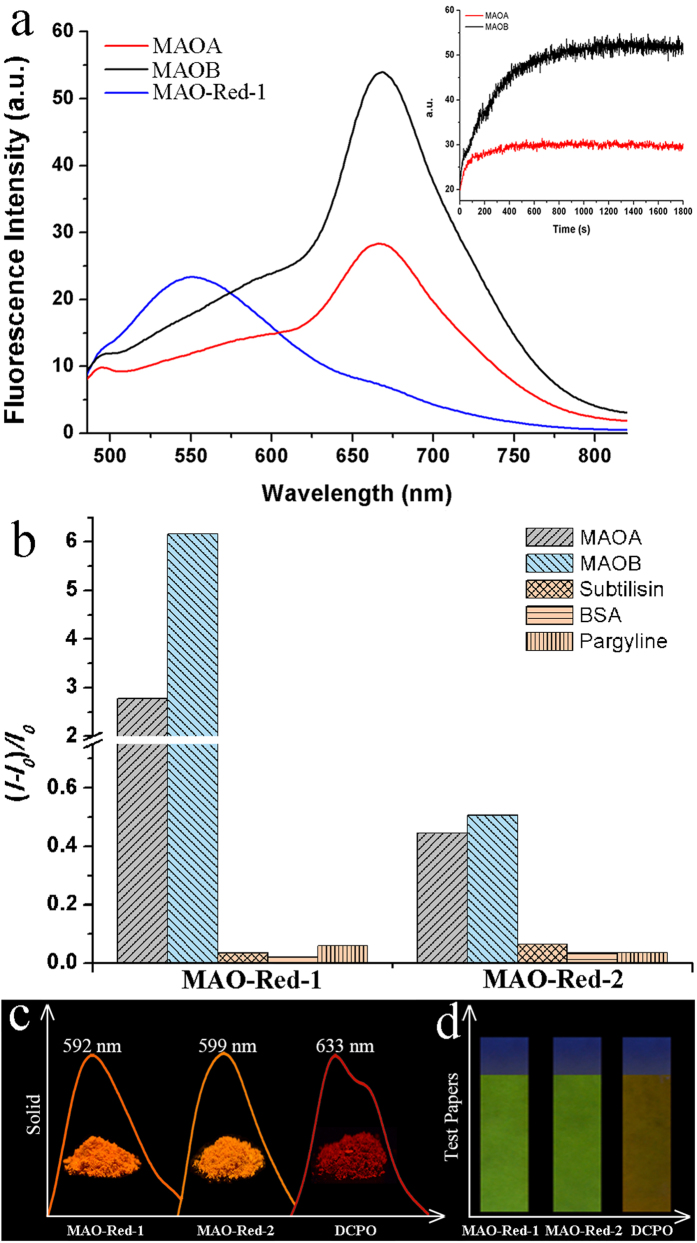
Fluorescent properties of **MAO-Red-1** and **MAO-Red-2**. (**a**) Fluorescence intensity of **MAO-Red-1** before and after reaction with MAO-A and MAO-B. Inserted plot: fluorescence kinetic assay of **MAO-Red-1** (10 μM) after the addition of MAO (10 μg mL^−1^); (**b**) The signal-to-background ratio of **MAO-Red-1** and **MAO-Red-2** after reactions with MAO-A, MAO-B, subtilisin, BSA and inhibited MAO; [protein] = 0.01–0.15 mg mL^−1^. The data were recorded in enzyme assay buffer (100 mM HEPES, pH = 7.4 with 5% glycerol and 1% DMSO) at 37 °C (λ_ex_/λ_em_ = 420/664 nm); (**c**) Normalized fluorescent spectra of **MAO-Red-1**, **MAO-Red-2** and DCPO in the solid state; (**d**) Paper imaging of **MAO-Red-1**, **MAO-Red-2** and DCPO under 365 nm UV light.

**Figure 3:**
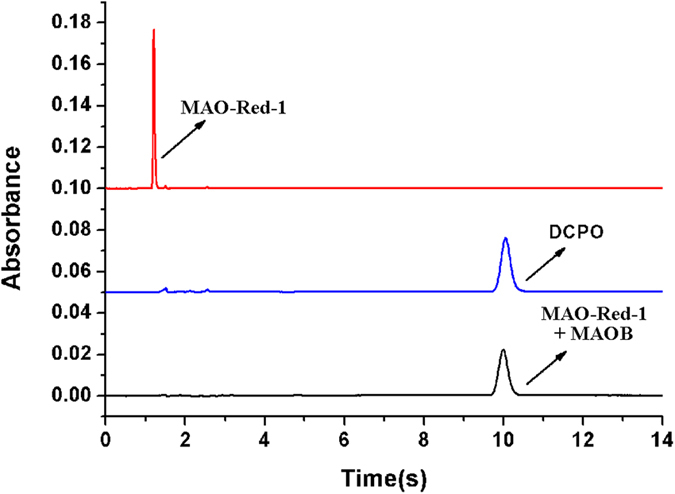
HPLC spectra of **MAO-Red-1**, DCPO, and the reaction product of **MAO-Red-1** and MAO-B.

To further prove the proposed MAO catalytic mechanism as stated in Fig. 1, **MAO-Red-1** (10 μM) was incubated with MAO-B (10 μg mL^−1^) in enzyme assay buffer (100 mM HEPES, pH 7.4 with 5% glycerol and 1% DMSO) at 37 °C for 1 hour. The reaction solution was then lyophilized, extracted with methanol, and tested on HPLC (Fig. 3). The cleavage product of **MAO-Red-1** and DCPO displayed the same retention time, conforming they were of the same substance. We also verified the result using high resolution mass spectra, the characteristic peak at 625.1429 (*m/z*) in HRMS spectra was correspond to [DCPO + H]^+^ (Fig. S4). Thus, we could safely say that the cleavage product was DCPO.

### Intracellular imaging of MAO

After demonstrating remarkable responsiveness and prominent selectivity towards MAO *in vitro*, we explored the possibility of applying our probes *in vivo*. Prior to imaging, it was essential for us to first validate the cytotoxicity of **MAO-Red-1** using MTS reduction assay. HeLa cells were incubated with various concentrations of **MAO-Red-1** (1, 1.25, 2, 2.5, 4, 5, 8, 10 μM) in 96-well plates for 24 h, the cell proliferation assay kit were then deployed according to standard procedure. Given the fact that above 80% of cells were still alive after treated with the maximum test concentration (10 μM) of **MAO-Red-1** (Fig. S5), the toxicity of **MAO-Red-1** is therefore neglectable.

Following that, we examined the photostability of **MAO-Red-1** inside mammalian cells, which was a crucial factor for the successful imaging of MAO. Since MAO is a mitochondrial enzyme, we chose a commercially available Mito-Tracker Green as the comparative dye to co-stain HeLa cells with **MAO-Red-1** for a total incubation time of 1 hour. The cells were then exposed to 405 nm (20 mW) and 588 nm (20 mW) channels of the solid state lasers, respectively. By the time of four minutes, the signal loss for Mito-tracker Green reached 91% while only 6% for MAO-Red-1 and 5% for DCPO (Fig. S6). Thus far, the photo stability of **MAO-Red-1** is well proved both *in vitro* and *in vivo*.

**Figure 4:**
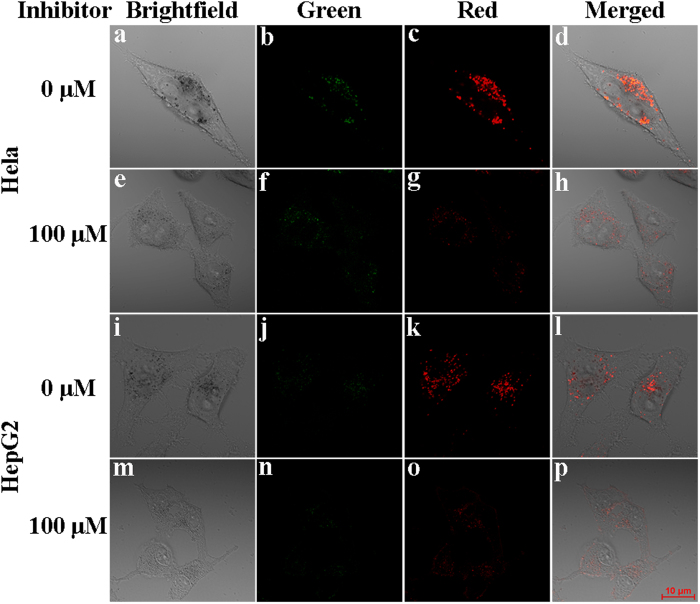
The Confocal fluorescence images of **MAO-Red-1** in HeLa and HepG2 cells with or without the presence of MAO-B inhibitor Pargyline. (**a,e,i,m**) Bright field images of HeLa and HepG2 cells incubated with **MAO-Red-1** (5 μM) for 1 h; (**b,f,j,n**) fluorescence images from Green channel (λ_ex_ = 405 nm); (**c,g,k,o**) fluorescence images from red channel (λ_ex_ = 405 nm); (**e–h,m–p**) fluorescence images of cells that was incubated with pargyline for 2 h followed by 1 h incubation of **MAO-Red-1**. The fluorescence images were collected at 420–520 nm (green channel) and 630–700 nm (red channel), respectively.

Subsequently, we studied the cell imaging ability of **MAO-Red-1**. HeLa and HepG2 cells were chosen in this study on account of their positive expression of MAO. For the purpose of discussing the sensitivity of **MAO-Red-1** in reporting MAO activities, both HeLa and HepG2 cells were divided into two groups (i.e. Group 1 and Group 2). Group 1 was incubated with 5 μM **MAO-Red-1** for 1 h at 37 °C, while Group 2 was first treated with 100 μM MAO inhibitor pargyline for 2 h at 37 °C. Afterwards, **MAO-Red-1** was added to Group 2 with the presence of pargyline, when the incubation time reached 1 h, the growth medium was removed and both groups were washed twice with PBS (PH = 7.4) to make sure no extracellular dye residue were left. The confocal imaging experiment was then carried out under dual-emission mode (λ_ex_ = 405 nm), while no obvious change were observed in green channel, the red channel displayed distinct variation. The cells that were only treated with **MAO-Red-1** showed significant red fluorescence, while the other cells that were pre-treated with MAO-B inhibitor pargyline exhibited very weak fluorescence (Fig. 4). This phenomenon was congruent with the previous carried out *in vitro* experiments, since the intensity at 550 nm (green channel) slightly decreased and a dramatic increase was observed at around 664 nm (red channel) after the addition of MAO.

The successful imaging of MAO in living cells inspired us to further explore the intracellular staining abilities of **MAO-Red-1** quantitatively via flow cytometry. As illustrated in Fig. S7, 90% cells that were pretreated with **MAO-Red-1** displayed stronger fluorescence compared with untreated cells, while only 18% pargyline and **MAO-Red-1** co-incubated cells exhibited the same increase. These results further demonstrated that **MAO-Red-1** was adequate for intracellular detection of MAO activities.

## Conclusion

In summary, we had designed and synthesized two red emission fluorescent probes with high sensitivity and selectivity towards MAO. *In vitro*, our probes successfully accomplished the identification of MAO on test papers and the detection limit demonstrated by **MAO-Red-1** was relatively low compared with previously published MAO probes. The cleavage product was unequivocally conformed by HPLC and LCMS. *In vivo,* not only did the intracellular experiments validate the low cytotoxicity and high photostability of **MAO-Red-1,** but it was proven both in HeLa and HepG2 cells that **MAO-Red-1** was capable of reporting MAO activities. Moreover, the CLSM experiments and flow cytometric studies jointly proved that **MAO-Red-1**could providea sensitive and accurate molecular tool for MAO imaging in complicated intracellular environment.

## Methods

### Chemicals and equipments

Unless otherwise stated, all reagents and solvents were obtained from commercial suppliers, and were used without further purification. Column chromatography was performed on silica gel (Qingdao haiyang) 300–400 mesh. All solvents used in spectra test systems were chromatographically pure. MAO-B inhibitor, Pargyline hydrochloride (P8013), human recombinant Monoamine Oxidase A (M7316) and B (M7441) (5 mg/ml) were purchased from Sigma Aldrich. Ultrapure water was used throughout.

^1^H NMR and ^13^C NMR spectra were recorded on a Bruker AMX-400 with chemical shifts expressed in parts per million (in deuteriochloroform or DMSO-d_6_, Me_4_Si as internal standard). UV−vis absorption spectra were recorded on a Hitachi PharmaSpec UV-1900 UV−visible spectrophotometer. Fluorescence spectra were determined using a HITAI F-7000 Spectro fluorophotometer. Absolute quantum yields were collected on a Horiba Jobin Yvon-Edison Fluoromax-4 fluorescence spectrometer with a calibrated integrating sphere system. To reduce the fluctuation in the excitation intensity, the xenon lamp was kept on for 1 hour prior to the experiment. The photostability experiments were performed on a Leica TCS SP8 confocal fluorescent microscope. The power percentages were both adjusted to 16.1%, the power on the focal plane were all 89 μW measured by a COHERENT LaserCheck power meter. The High-resolution mass spectra were obtained on a Finnigan LCQDECA and a Bruker Daltonics Bio TOF mass spectrometer. TLC analyses were performed on silica gel GF 254 using UV light as visualizing agent. The pH values were determined by a Leici pH3c (digital display) pH meter.

### Synthetic procedures

The synthetic procedures are shown in Fig. 1b. All of the NMR and LCMS spectra for the corresponding products are shown in the supplementary information.

### 1-tert-Butoxycarbonylamino-3-bromopropane (S1)

A solution of 4-dimethylaminopyridine (244.3 mg, 2 mmol) and riethylamine (1.4 mL, 10 mmol) in dichloromethane (10 mL) was added dropwise to a suspension of 3-bromopropylamine hydrobromide (2.2 g, 10 mmol) and di-tert-butyl dicarbonate (2.3 g, 10.5 mmol) in dichloromethane (50 mL), then the mixture was stirred at room temperature for another 30 minutes to become a transparent solution. After that, the solution was washed successively with 0.5N HCl aq., 5 wt % of NaHCO_3_ aq., and brine, and then dried with NaSO_4_. After filtration, the solvent was removed under reduced pressure. **S1** was obtained as colorless oil (2.3 g, 97% yield) and was then used without further purification.

### (E)-2-(2-(4-methoxystyryl)-4H-chromen-4-ylidene) malononitrile (S2)

2-(2-methyl-4H-chromen-4-ylidene) malononitrile (1 g, 4.8 mmol) and 4-methoxybenzaldehyde (0.58 mL, 4.8 mmol) was dissolved in a mixture of ethanol and toluene (100 mL/40 mL), 20 drops of piperidine was added to the mixture. The solution was heated to reflux for 12 hours, and then the solvent was removed under reduced pressure. The crude product was then purified by chromatography on silica gel (petrol ether: dichloromethane, from 50:1 to 3:1, v/v) to afford **S2** as a red solid. ^1^H NMR (400 MHz, CDCl_3_) δ 8.94 (d, J = 8.4 Hz, 1 H), 7.76 (m, *J* = 8.4 Hz, 1 H), 7.65–7.56 (m, 4 H), 7.47 (m, *J* = 8.2 Hz, 1 H), 6.99 (d, J = 8.6 Hz, 2 H), 6.85 (s, 1 H), 6.71 (d, *J* = 15.8 Hz, 1 H), 3.90 (s, 3 H);^13^C NMR (101 MHz, CDCl_3_) δ 161.7, 158.0, 152.8, 152.3, 138.7, 134.5, 129.7, 127.3, 125.8, 125.7, 118.5, 117.8, 116.9, 116.2, 115.9, 114.6, 106.1, 61.9, 55.4; HRMS (ESI) calcd.for C_21_H_14_N_2_O_2_, [M + H]^+^: 327.1134; found: 327.1109.

### (E)-2-(2-(4-hydroxystyryl)-4H-chromen-4-ylidene) malononitrile (S3)

BBr_3_ (1.86 mL, 20 mmol) was diluted with anhydrous dichloromethane (30 mL), then was added dropwise over a period of 3 hours at 0 °C to the solution of **S2** (1.3 g, 4 mmol) in 100 mL anhydrous dichloromethane. The mixture was allowed to warm up to room temperature and stirred overnight under nitrogen protection, after that the reaction was quenched with 10 mL water, the generated solid was filtered and washed with ethyl acetate. The filtrate was extracted with EtOAC (100 mL × 3); the organic layer was dried over NaSO_4_. The solvent was removed by rotary evaporation and the crude product was then purified by chromatography on silica gel (petrol ether: dichloromethane, from 10:1 to 0:1, v/v) furnished **S3** as a red solid. ^1^H NMR (400 MHz, d_6_-DMSO) δ 10.14 (s, 1 H), 8.68 (dd, *J* = 8.4, 1.2 Hz, 1 H), 7.88 (m, 1 H), 7.75–7.72 (m, 1 H), 7.65–7.54 (m, 4 H), 7.21 (d, *J* = 15.9 Hz, 1 H), 6.89–6.83 (t, 3 H); ^13^C NMR (101 MHz, CDCl_3_) δ 165.2, 164.0, 157.9, 157.1, 144.4, 140.4, 135.5, 131.3, 131.2, 129.7, 124.1, 122.5, 122.2, 121.2, 121.0, 110.8, 64.3; HRMS (ESI) calcd. for C_20_H_12_N_2_O_2_, [M^—^H]^−^: 311.0826; found: 311.0827.

### (E)-tert-butyl (3-(4-(2-(4-(dicyanomethylene)-4H-chromen-2-yl) vinyl) phenoxy) propyl) carbamate (S4)

To a stirred suspension of **S3** (200 mg, 0.64 mmol), and K_2_CO_3_ (138.2 mg, 1 mmol) in DMF (10 mL), **S1** (166 mg, 0.7 mmol) was added. After stirred at room temperature for 10 to 12 hours, the reaction was quenched by water (100 mL), aqueous phase was extracted with EtOAC (100 mL × 3), the organic layer was dried over NaSO_4_. The solvent was removed by rotary evaporation and the crude product was then purified by chromatography on silica gel (petrol ether: dichloromethane, from 10:1 to 0:1, v/v) to afford **S4** as a red solid. ^1^H NMR (400 MHz, CDCl_3_) δ 8.91 (dd, *J* = 8.4, 1.2 Hz, 1 H), 7.73 (m, 1 H), 7.60–7.52 (m, 4 H), 7.44 (m, 1 H), 6.95 (d, *J* = 8.8 Hz, 2 H), 6.83 (s, 1 H), 6.68 (d, *J* = 15.8 Hz, 1 H), 4.74 (s, 1 H), 4.08 (t, *J* = 6.0 Hz, 2 H), 3.35 (q, 2 H), 2.05–1.99 (m, 2 H), 1.45 (s, 9 H); ^13^C NMR (101 MHz, CDCl_3_) δ 160.9, 157.9, 156.0, 152.8, 152.3, 138.7, 134.5, 129.7, 127.4, 125.8, 125.7, 118.5, 117.8, 116.9, 116.2, 115.1, 106.2, 79.3, 65.9, 62.0, 37.8, 29.5, 28.4; HRMS (ESI) calcd. for C_28_H_27_N_3_O_4_, [M + Na]^+^: 492.1899; found: 492.1868.

### (E)-2-(2-(4-(3-aminopropoxy)styryl)-4H-chromen-4-ylidene) malononitrile (MAO-Red-1)

**S4** (249 mg, 0.53 mmol) was dissolved in 5 mL dichloromethane, and was then added dropwise to saturated hydrogen chloride methanol solution. After stirred at room temperature for 10 to 12 hours, the mixture was concentrated under reduced pressure giving **MAO-Red-1** (192 mg, 98%) as orange solid. ^1^H NMR (400 MHz, d_6_-DMSO) δ 8.68 (d, *J* = 8.2 Hz, 1 H), 8.04 (s, 3 H), 7.88 (t, *J* = 7.9 Hz, 1 H), 7.77–7.62 (m, 4 H), 7.56 (t, *J* = 7.6 Hz, 1 H), 7.31 (d, *J* = 15.8 Hz, 1 H), 7.00 (d, *J* = 8.4 Hz, 2 H), 6.92 (s, 1 H), 4.11 (t, *J* = 5.8 Hz, 2 H), 2.92 (t, *J* = 6.8 Hz, 2 H), 2.02 (m, 2 H); ^13^C NMR (101 MHz, d_6_-DMSO) δ 160.6, 158.9, 153.2, 152.3, 139.0, 135.7, 130.4, 128.1, 126.5, 125.0, 119.4, 117.6, 117.5, 117.4, 116.3, 115.5, 106.4, 65.3, 59.9, 36.5, 27.1; HRMS (ESI) calcd. for C_23_H_19_N_3_O_2_, [M + H]^+^: 370.1556; found: 370.1551.

### (E)-2-(2-(4-(3-(dimethylamino) propoxy) styryl)-4H-chromen-4-ylidene) malononitrile (MAO-Red-2)

This compound was prepared according to the same process for the synthesis of **S4**, Yield: 61%.^1^H NMR (400 MHz, d_6_-DMSO) δ 9.29 (s, 1 H), 8.69 (d, *J* = 8.2 Hz, 1 H), 7.89 (t, *J* = 7.6 Hz, 1 H), 7.83–7.63 (m, 4 H), 7.58 (t, *J* = 7.6 Hz, 1 H), 7.34 (d, *J* = 16.0 Hz, 1 H), 7.02 (d, *J* = 8.5 Hz, 2 H), 6.95 (s, 1 H), 4.10 (t, *J* = 5.7 Hz, 2 H), 3.25–3.16 (m, 2 H), 2.80 (s, 6 H), 2.08 (m, 2 H);^13^C NMR (101 MHz, d_6_-DMSO) δ 160.5, 158.9, 153.2, 152.4, 138.9, 135.7, 130.4, 128.3, 126.5, 125.0, 119.4, 117.7, 117.6, 117.5, 116.3, 115.5, 106.5, 65.4, 59.9, 54.6, 42.8, 24.3; HRMS (ESI) calcd. for C_25_H_23_N_3_O_2_, [M + H]^+^: 398.1869; found: 398.1870.HeLa cells were cultured in Dulbecco’s modified Eagle medium (DMEM) containing 10% fetal bovine serum and 1% antibiotic–antimycotic at 37 °C in a 5% CO_2_/95% air incubator. Fluorescence imaging, cells (4 × 10^3^ per well) were passed on confocal dishes and incubated for 24 h. Immediately before the staining experiments, the cells were washed twice with PBS (10 mM).

### Parameters for the HPLC tests

HPLC measurements were carried out on Waters e2695 Separatins Module using Waters 2998 PDA detector equipped with a Symmetry C18 column (4.6 × 250 mm, 5 μm). Water (A) and methol (B) were used as eluents, the column was gradient eluted (A: B = 95-5%) for 20 minutes with a flow rate of 1 ml/min. 0.1%TFA was added to solvent A. 365 nm was used as the excitation wavelength.

### Procedures for the cytotoxicity and confocal laser scanning microscopy (CLSM) experiments

HEK293 cells (human embryonic kidney) and HeLa cells were incubated in Dulbecco’s modified Eagle’s medium (DMEM) containing 10% fetal bovine serum (FBS) and 1% antibiotics (penicillin–streptomycin, 10,000 U mL^−1^) at 37 °C in a humidified atmosphere containing 5% CO_2_.

Toxicities of **MAO-Red-1** toward HeLa cells were determined by using MTS reduction assay following literature procedures. About 1.0 × 10^4^ cells/well was seeded into 96-well plates. After 24 h, various concentrations of **MAO-Red-1** (1, 1.25, 2, 2.5, 4, 5, 8, 10 μM) were added to the cells. After another 24 h, 20 μL MTS and 100 μL PBS were added to each well and the plates were incubated at 37 °C for another 1 h. Then the absorbance of each sample was measured using an ELISA plate reader (model 680, BioRad) at a wavelength of 490 nm. The cell viability (%) was obtained according to the manufacturer’s instruction.

For the confocal laser scanning microscopy (CLSM) experiments, HEK 293 cells and HeLa cells were seeded at a density of 2.5 × 10^5^ cells per well in 35 mm confocal dish (Φ = 15 mm), 24 h before the addition of **MAO-Red-1** or pargyline followed by **MAO-Red-1**. The cells were observed with a ZEISS LSM 780 confocal laser scanning microscope.

### Flow Cytometric Study

HeLa cells were seeded in 12-well plates (2.0 × 10^5^ cells/well) and allowed to attach and grow for 24 h. Then the cells were treated with **MAO-Red-1** (5 μM) or pargyline (100 μM, 2 h) followed by **MAO-Red-1** (5 μM) in triplicate. Subsequently, the cells were washed with 1 × PBS, trypsinized, centrifuged and washed twice with 1 × PBS. The cell pellets were then resuspended. Mean fluorescence intensity was analyzed using the flow cytometer (BD Accuri C6) under the λ_ex_/λ_em_ mode (FL3 Red channel: filter 695 ± 40 nm, λ_ex_: 488 nm).

## Additional Information

**How to cite this article**: Li, L.-L. *et al.* Red emission fluorescent probes for visualization of monoamine oxidase in living cells. *Sci. Rep.* **6**, 31217; doi: 10.1038/srep31217 (2016).

## Supplementary Material

Supplementary Information
