# Supplementary material for: Red emission fluorescent probes for visualization of monoamine oxidase in living cells
Source: Sci Rep. 2016 Aug 8;6:31217. doi: 10.1038/srep31217 (PMC4976310; doi:10.1038/srep31217)
Supplement: Supplementary Information [file srep31217-s1.pdf]

Supporting Information

Red emission fluorescent probes for visualization of monoamine oxidase in living cells

Ling-Ling Li, Kun Li*,Yan-Hong Liu, Hao-Ran Xu and Xiao-Qi Yu*

Key Laboratory of Green Chemistry and Technology (Ministry of Education), College ofChemistry, Sichuan University, Chengdu, 610064, P.R. China.

Contents

1.Limit of detection S1

2.Absorption spectra S1

3.Fluorescence spectroscopy S2

4.HRMS characterization of the enzymatic reaction product between MAO-Red-1 and MAO-B S2

5.Enzymatic kinetics assays S2

6.Cytotoxicity experiments and Confocal laser scanning microscopy (CLSM) S3

7.Intracellular photostability S3

8.The histogram of flow cytometry in Hela cells S4

9.NMR and HRMS spectra of the synthetic products S5

1. Limit of detection

The limit of detection, expressed as the concentration, CL, CL =3σ/m

$$\sigma=\sqrt{\frac{\Sigma(\bar{x}-x_i)^2}{n-1}}$$

$\bar{x}$is the mean of the blank measures (probe only), $x_i$is the values of blank measures, n is the tested number of blank measure, m is the slope of the linear regression equation.

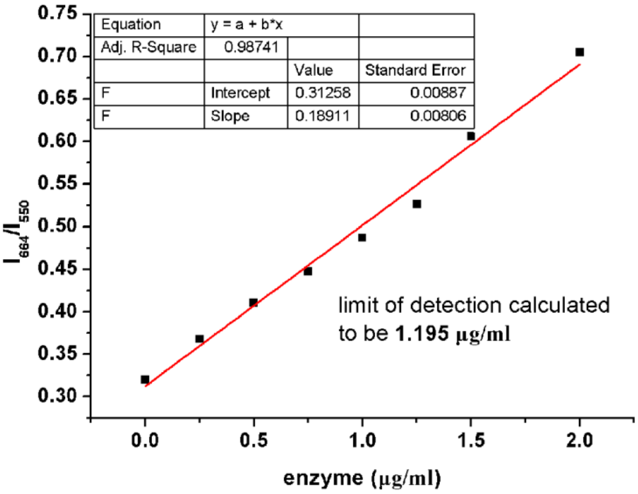

Figure S1. The line relationship between the fluorescent intensity ratio of **MAO-Red-1** (10 µM at 550 nm and 664 nm) and the concentration of MAO-B at 0 to 2 µg ml^{-1} inenzyme assay buffer (100 mM HEPES, pH=7.4 with 5% glycerol and 1% DMSO) at 37 ^{o}C.

2. Absorption spectra

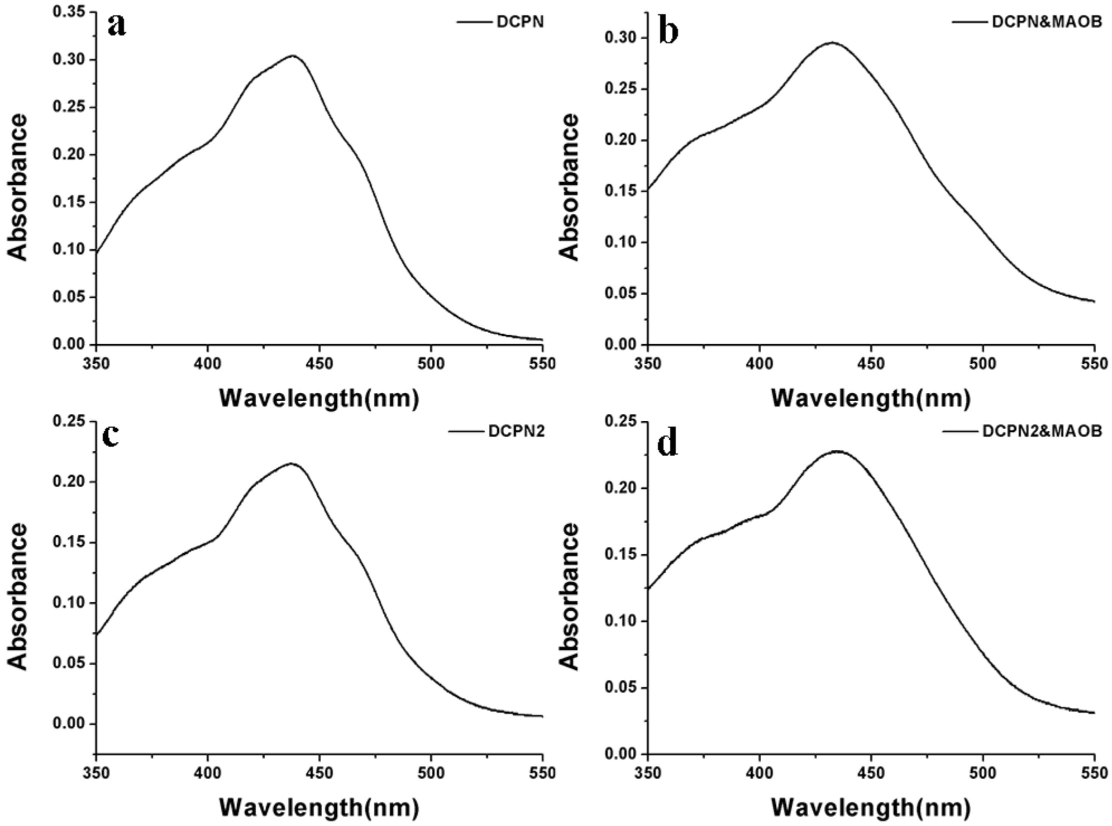

Figure S2. Absorption spectra of (a) **MAO-Red-1** (10 μM); (b) **MAO-Red-1** (10 μM) with MAOB (10 μg/ mL);(c) **MAO-Red-2** (10 μM); (d) **MAO-Red-2** (10 μM) with MAOB (10 μg/ mL) inenzyme assay buffer (100 mM HEPES, pH=7.4 with 5% glycerol and 1% DMSO) at 37°C.

3. Fluorescence spectroscopy

The detection of activity of enzymes with Probe were performed in enzyme assay buffer (100 mM HEPES, pH=7.4 with 5% glycerol and 1% DMSO) at 37℃, all fluorescence data were collected at $\lambda_{ex}/\lambda_{em}$ =420 /664 nm.

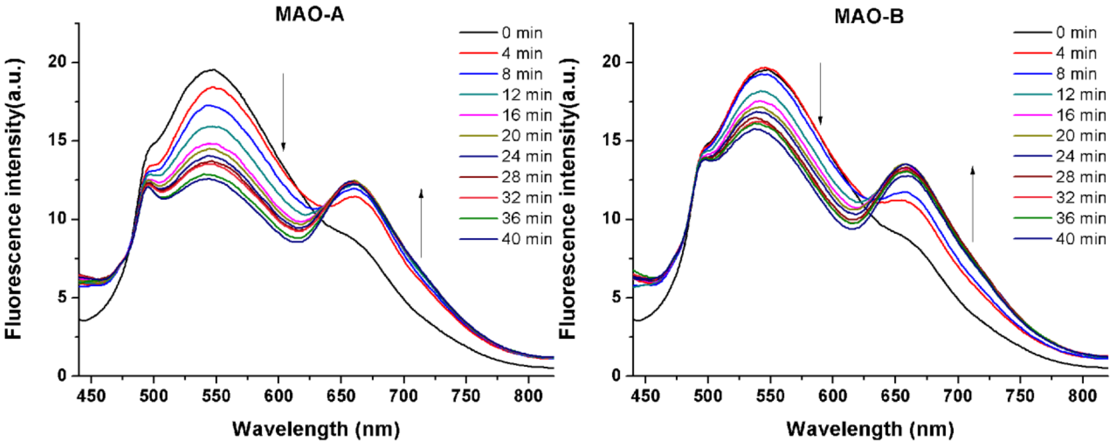

Figure S3. Time-dependent fluorescence changes of **MAO-Red-2** with MAO-A or MAO-B. λ_{ex} =420 nm.

4.HRMS characterization of the enzymatic reactionproduct between MAO-Red-1 and MAO-B.

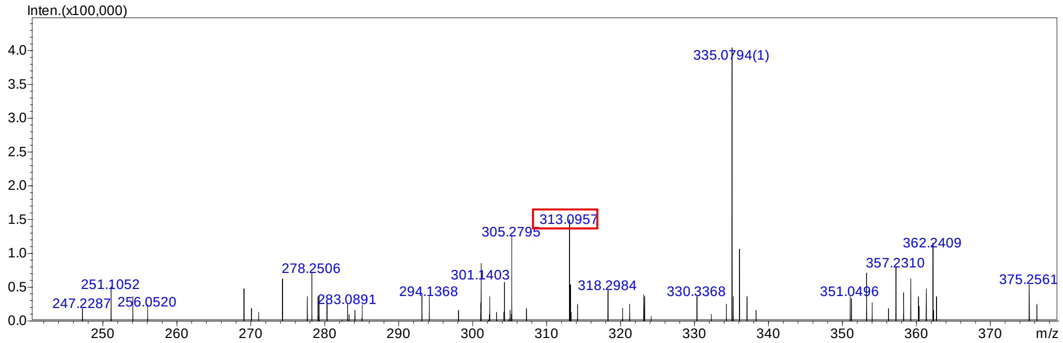

Figure S4. LC-MS result of the reaction product of **MAO-Red-1** and MAO-B.

5. Enzymatic kinetics assays

The enzymatic kinetic parameters for **MAO-Red-1**with MAO-B were calculated according to previous literature [1].

	K_m (μM)	V_{max} (nmol min^{-1} mg^{-1})
**MAO-Red-1 with MAO-B**	**270**	**23.2**

Table S1. The enzymatic kinetic parameters for **MAO-Red-1** with MAO-B

6. Cytotoxicity experiments and Confocal laser scanning microscopy (CLSM).

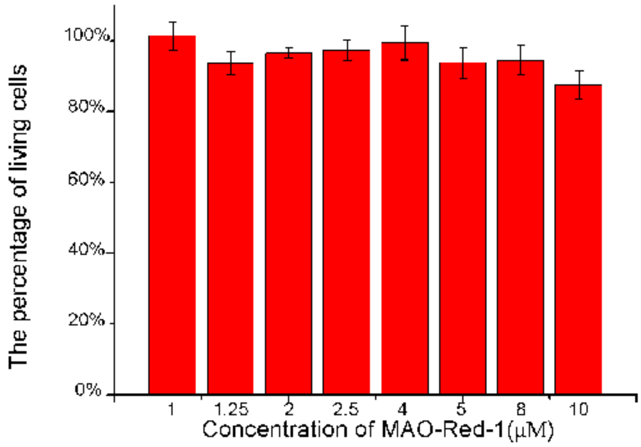

Figure S5. Cytotoxicity of differente concentrations of **MAO-Red-1** to Hela cells by a standard aqueous one solution cell proliferation assay, the experiment was repeated three times and the data are shown as mean (±S.D.).

7. Intracellular photostability.

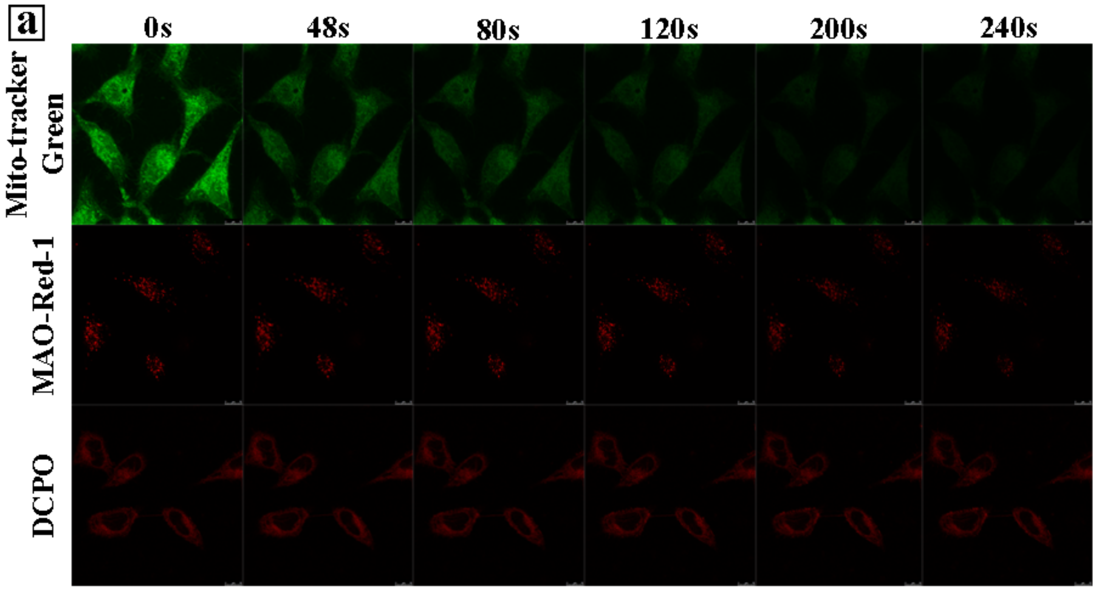

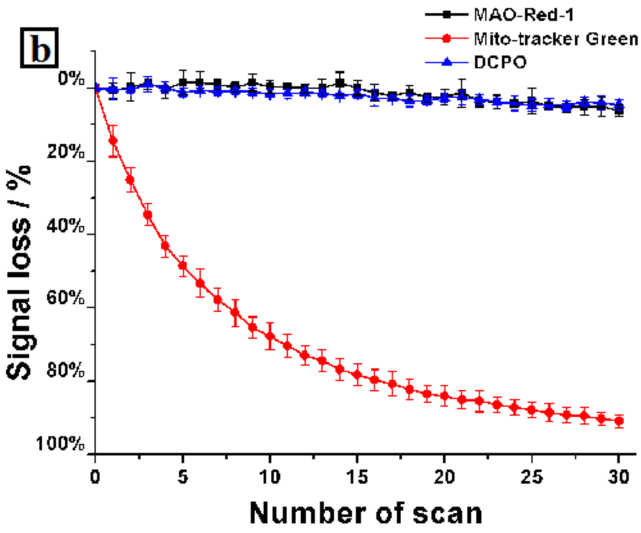

Figure S6. (a) Intracellular photostability images of Mito-tracker Green,**MAO-Red-1**and DCPO in Hela cells. **(b)** Signal loss (%) of fluorescent emission of **MAO-Red-1**, DCPO, and Mito-tracker Green in Hela cells with increasing number of scans using confocal microscope(irradiation time: 7.71 s/scan). For **MAO-Red-1** and DCPO:λ_{ex}= 405 nm, λ_{em}= 640−700 nm;for Mito-tracker Green: λ_{ex}= 488 nm, λ_{em}= 530-590nm. The power on the focal planewas 89μW.

8. The histogram of flow cytometry in Hela cells.

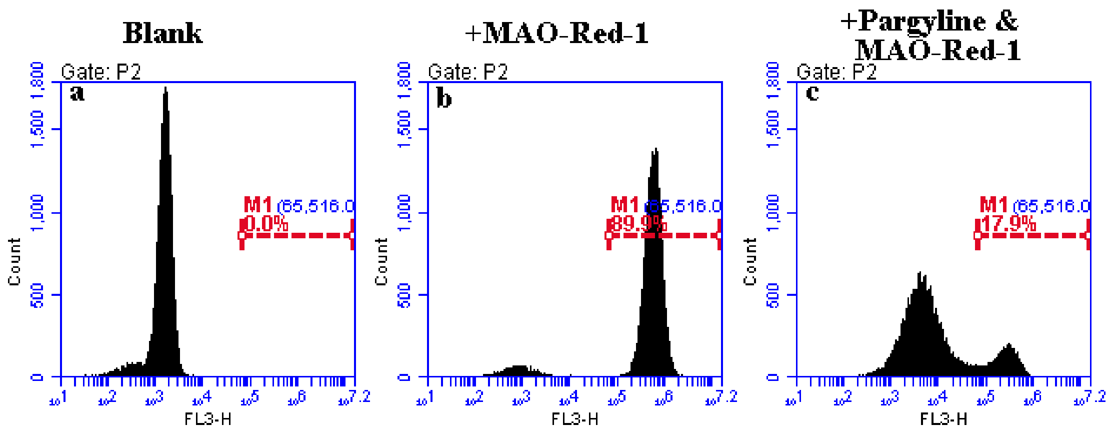

Figure S7. The histogram of flow cytometry of (a): Hela cells from the FL3 Red channel; (b) Hela cells stained with **MAO-Red-1** (5μM) from the FL3 Red channel; (c): Hela cells that were incubated with pargyline for 2h followed by 1 hour's incubation of **MAO-Red-1** (5μM). FL3 Red channel: filter 695 ±40 nm, λ_{ex} =488 nm.

9. NMR and HRMS spectra of synthetic products

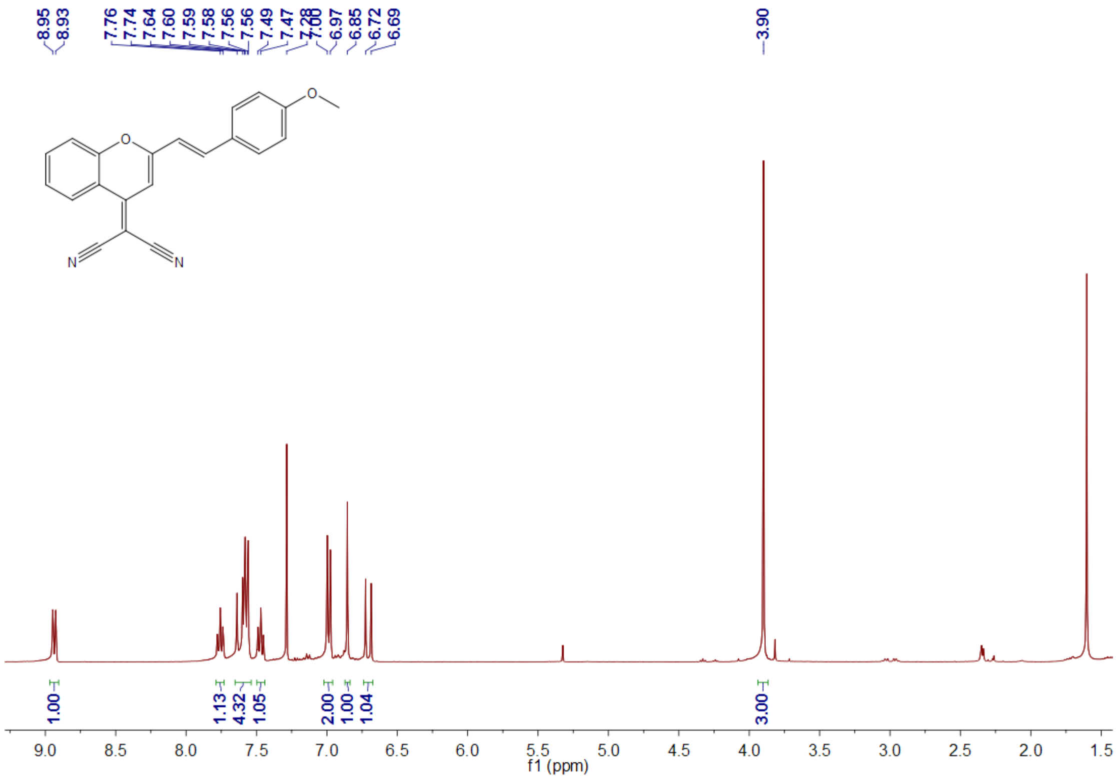

Figure S8. ^{1}H NMR spectrum of **S2** in $CDCl_3$.

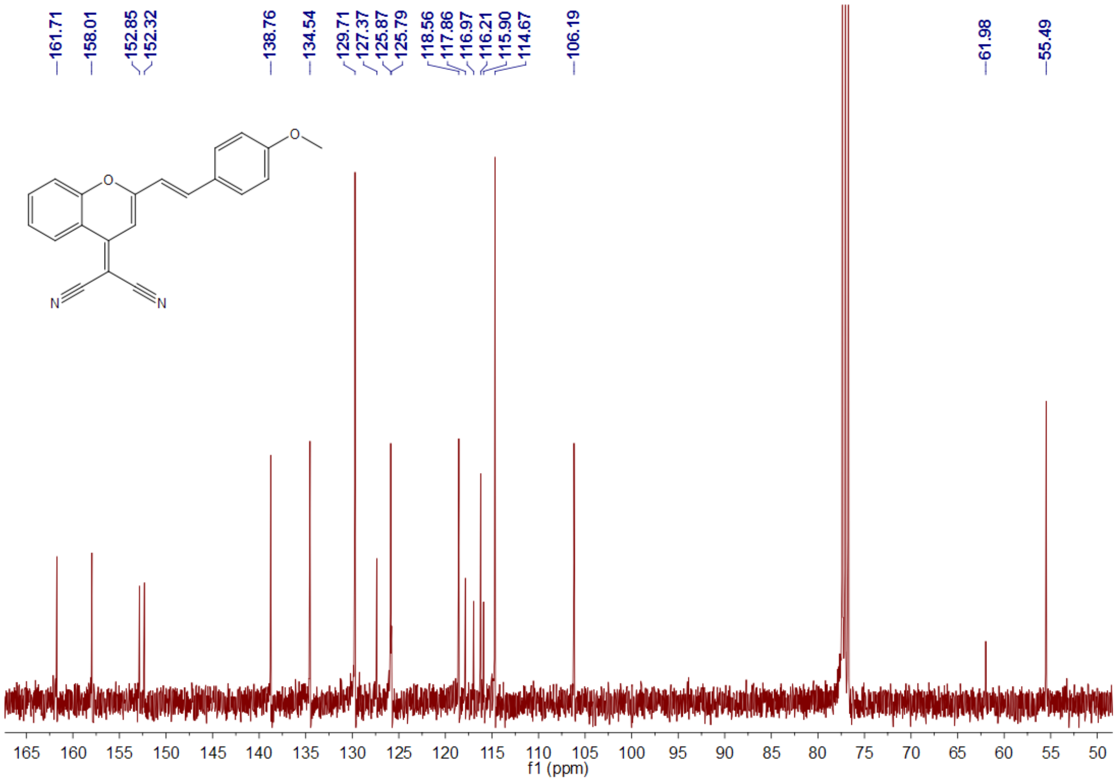

Figure S9. ^{13}C NMR spectrum of **S2** in $CDCl_3$.

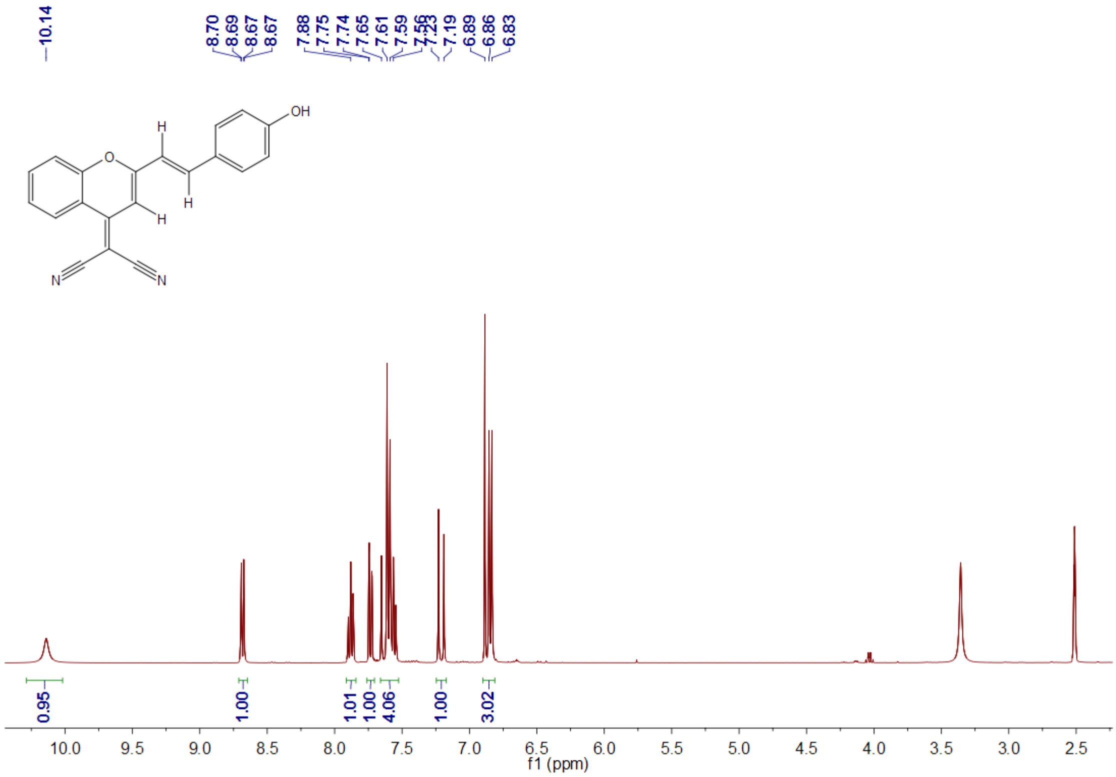

Figure S10. ^{1}H NMR spectrum of **S3** in d_6-DMSO.

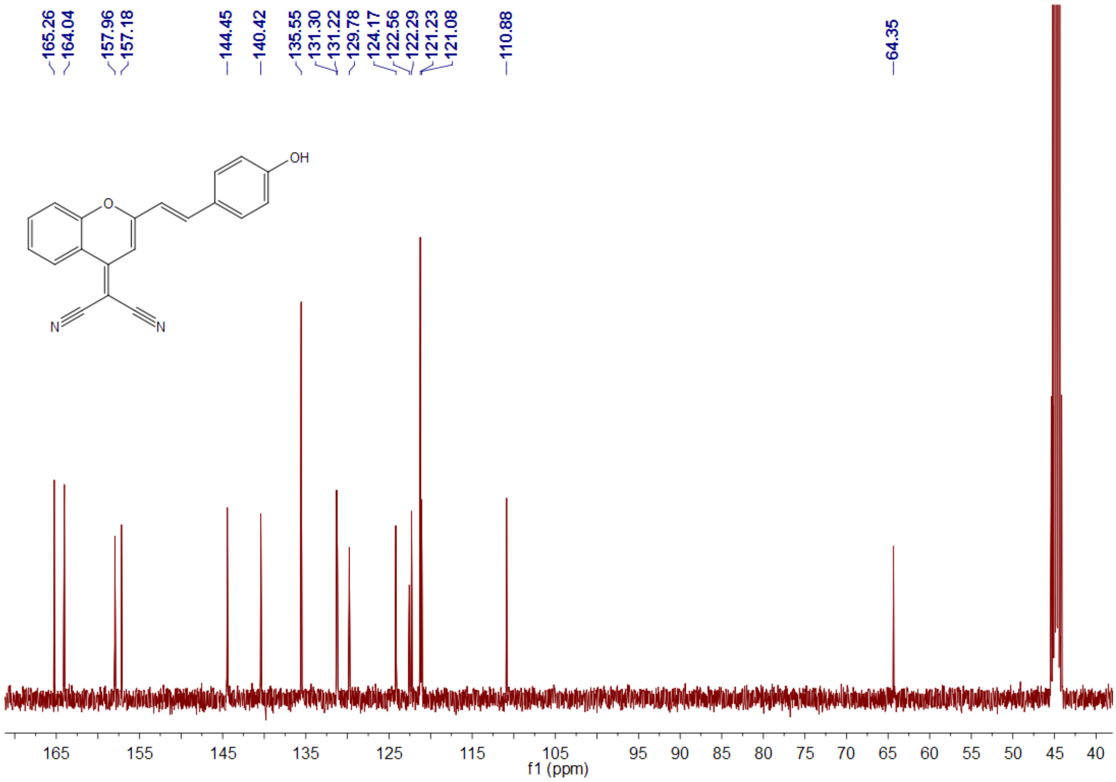

Figure S11. ^{13}C NMR spectrum of **S3** in d_6-DMSO.

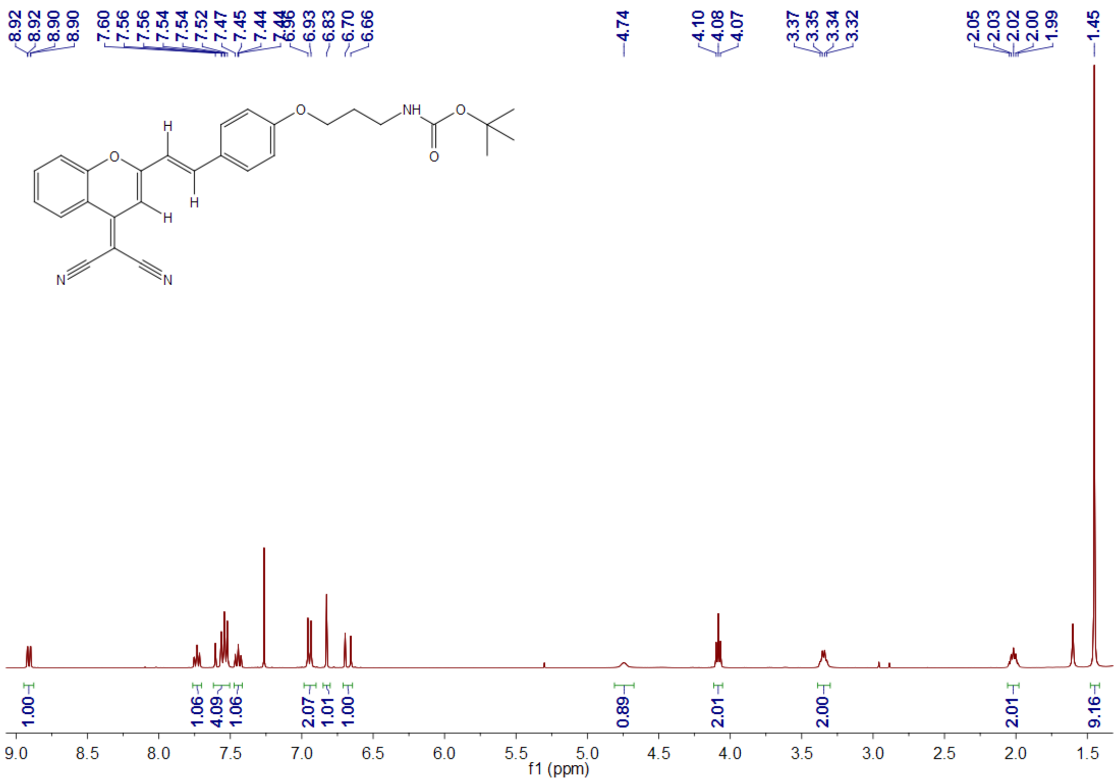

Figure S12. ^{1}H NMR spectrum of **S4** in $CDCl_3$.

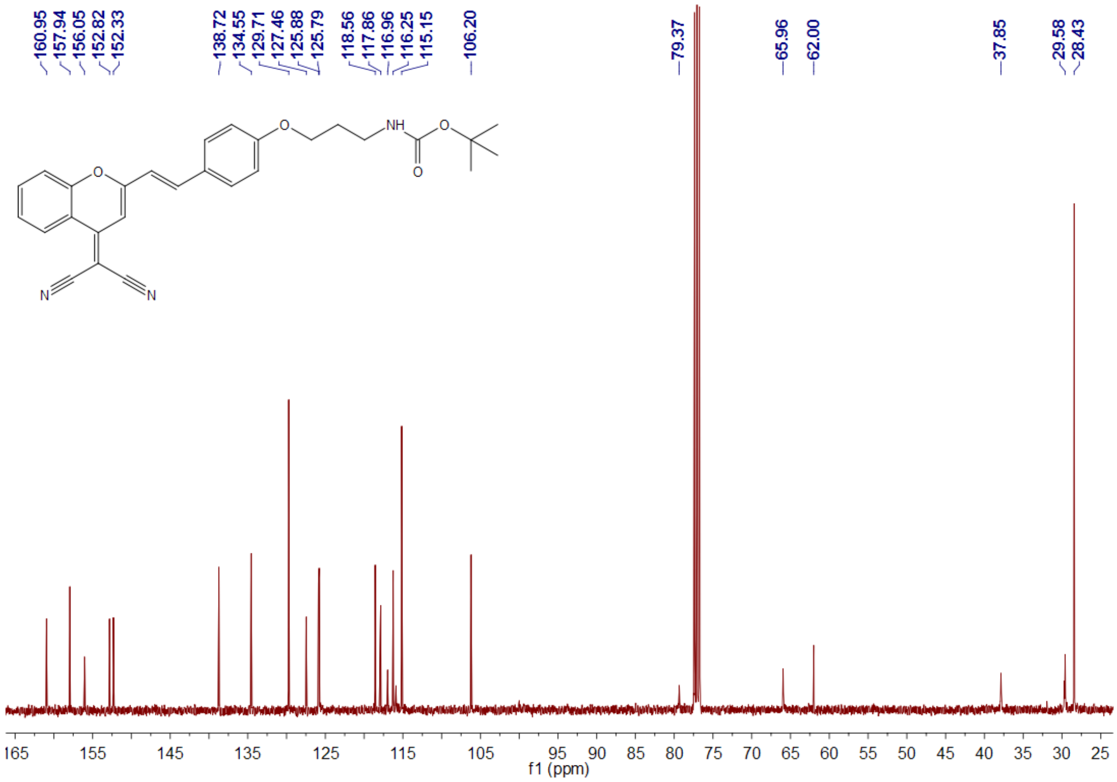

Figure S13. ^{13}C NMR spectrum of **S4** in $CDCl_3$.

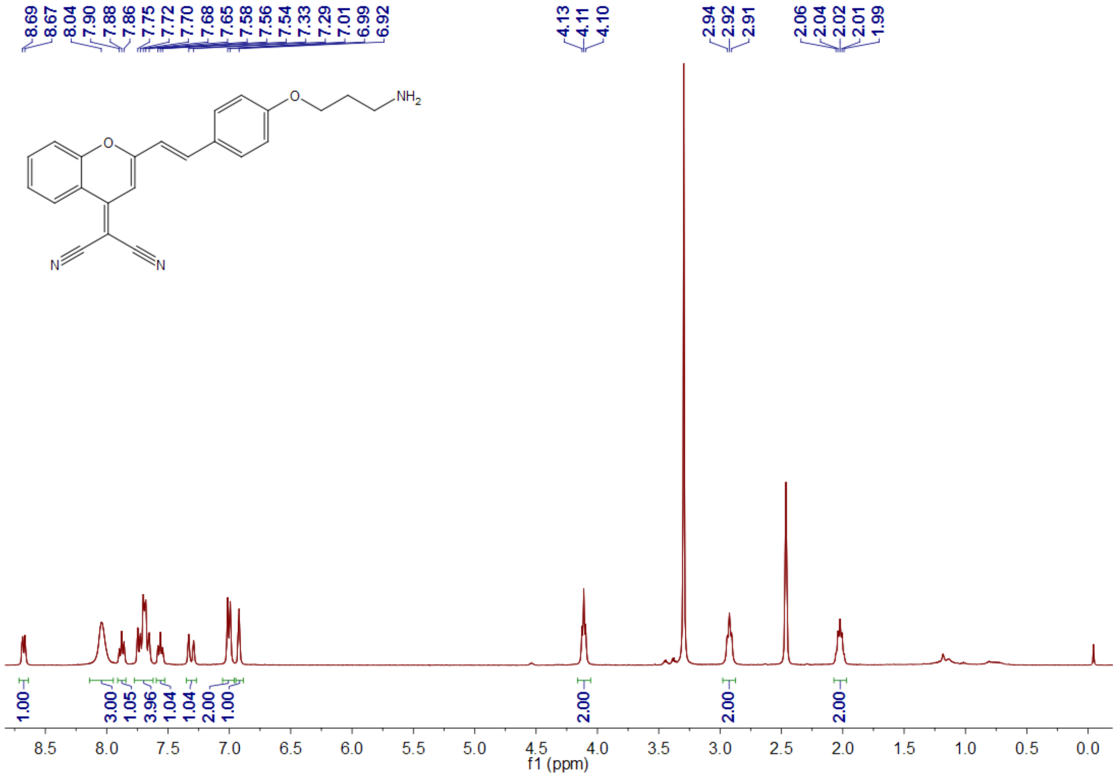

Figure S14. ^{1}H NMR spectrum of **MAO-Red-1** in d_6-DMSO.

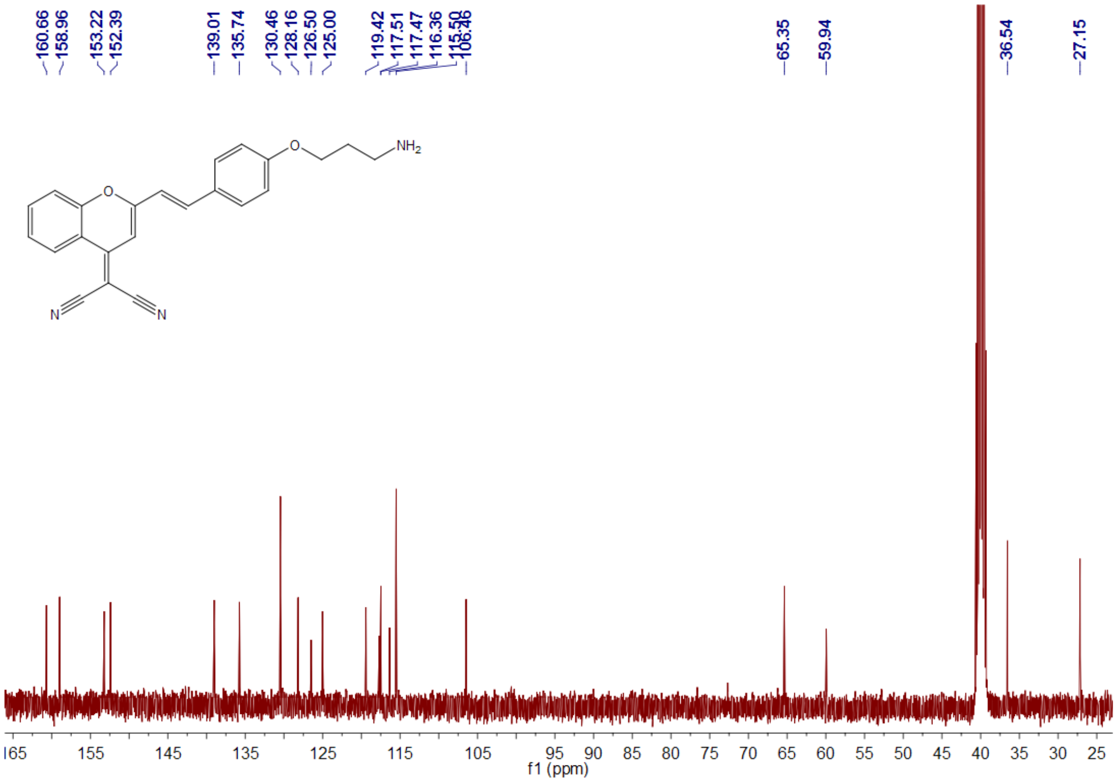

Figure S15. ^{13}C NMR spectrum of **MAO-Red-1** in d_6-DMSO.

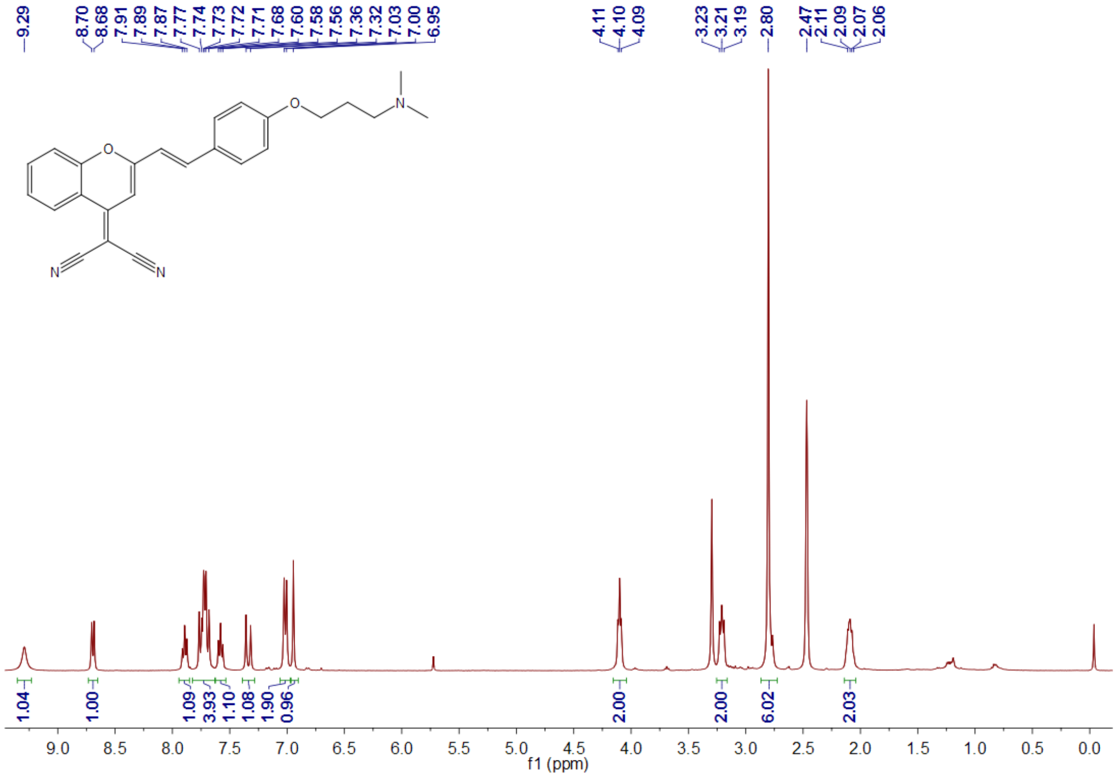

Figure S16. ^{1}H NMR spectrum of **MAO-Red-2** in d_6-DMSO

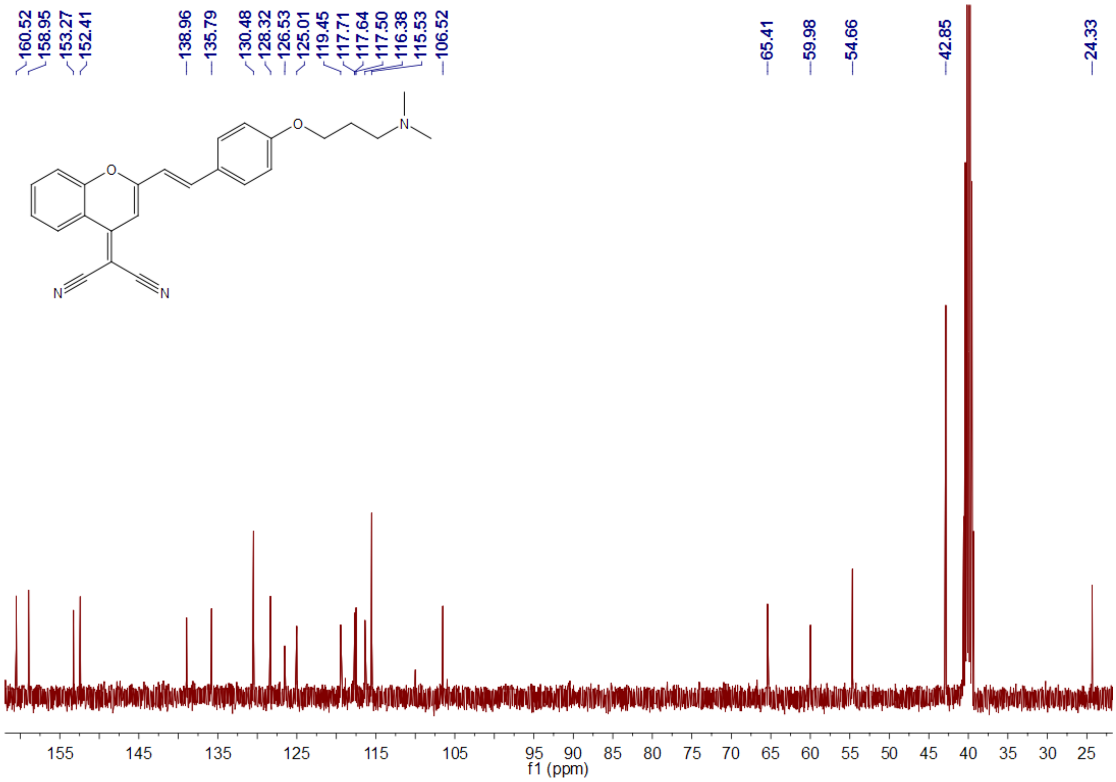

Figure S17. ^{13}C NMR spectrum of **MAO-Red-2** in d_6-DMSO

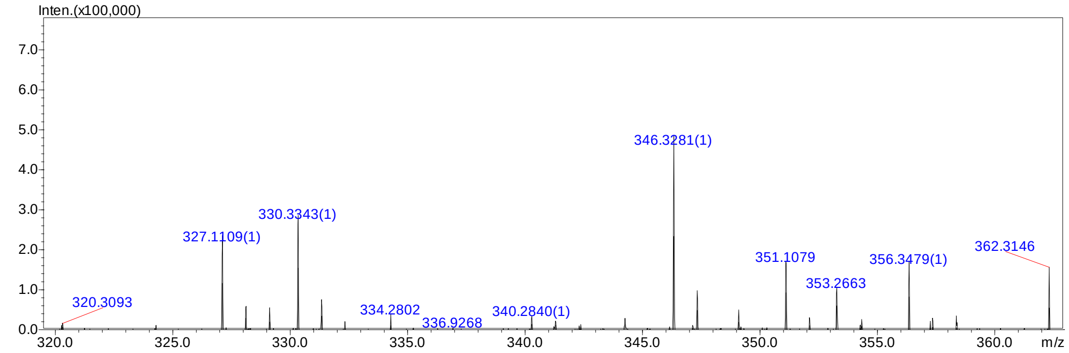

Figure S18. HRMS result of **S2**.

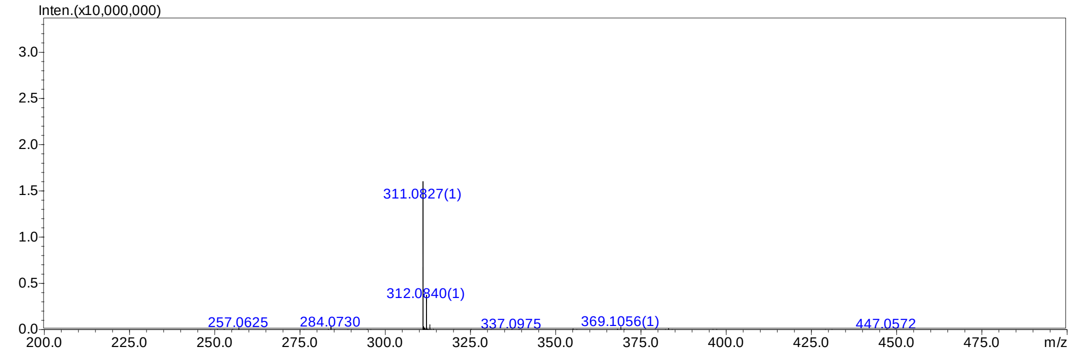

Figure S19. HRMS result of **S3**.

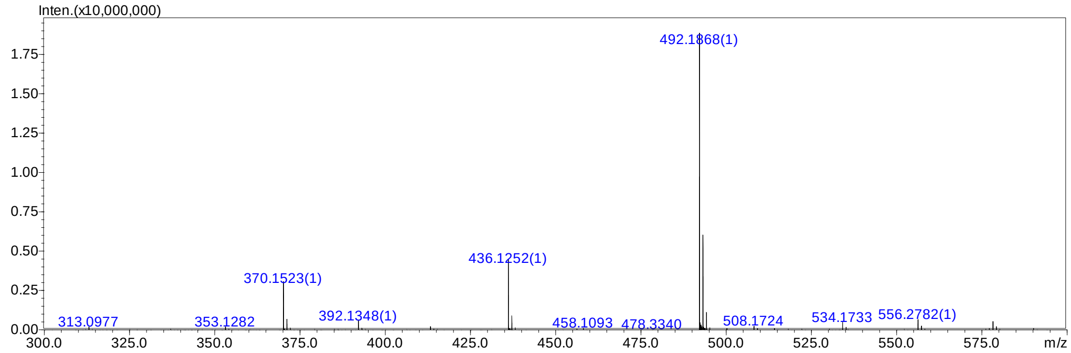

Figure S20. HRMS result of **S4**.

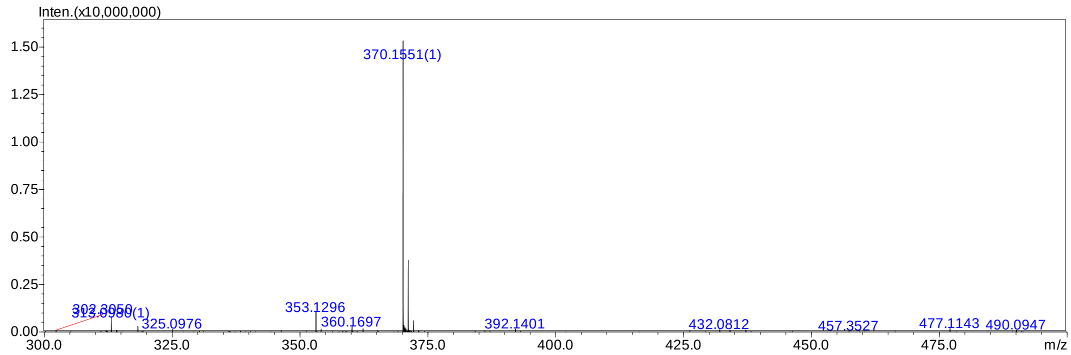

Figure S21. HRMS result of **MAO-Red-1**.

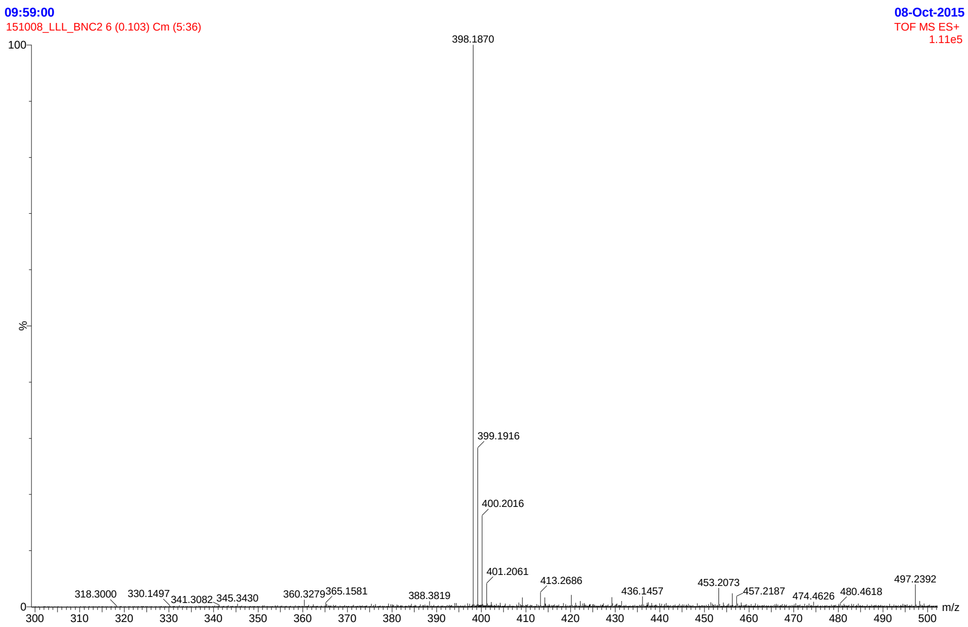

Figure S22. HRMS result of **MAO-Red-2**.

Reference

[1] Yamaguchi,K. et al. Design of Chemical Shift-Switching ^{19}F Magnetic Resonance Imaging Probe for Specific Detection of Human Monoamine Oxidase A. *J. Am. Chem. Soc.***133**, 14208–14211 (2011).
